# Abusive head trauma among children in Alaska: a population-based assessment

**DOI:** 10.3402/ijch.v72i0.21216

**Published:** 2013-08-05

**Authors:** Jared Parrish, Cathy Baldwin-Johnson, Margaret Volz, Yvonne Goldsmith

**Affiliations:** 1 MCH-Epidemiology Unit, Alaska Division of Public Health, Anchorage, AK, USA; 2 The Injury Prevention Research Center at the University of North Carolina, Chapel Hill, NC, USA; 3 The Children's Hospital at Providence, Anchorage, AK, USA

**Keywords:** child maltreatment, child abuse, abusive head trauma, traumatic brain injury, surveillance, epidemiology

## Abstract

**Background:**

Serious physical abuse resulting in a traumatic brain injury (TBI) has been implicated as an underreported cause of infant mortality. Nearly 80% of all abusive head trauma (AHT) occurs among children <2 years of age, with infants experiencing an incidence nearly 8 times that of 2-year olds.

**Objective:**

This study describes the validation of the CDC Pediatric Abusive Head Trauma (PAHT) definitions when applied to a multi-source database at the state level and provides a robust annual incidence estimate of AHT among children <2 years of age in Alaska.

**Design:**

AHT cases among children residing in Alaska during 2005–2010 were identified by applying the PAHT coding schema to a multi-source database which included vital death records, the Violent Death Reporting System (AK-VDRS), the Maternal Infant Mortality Review – Child Death Review (MIMR-CDR), the Alaska Trauma Registry (ATR), the inpatient Hospital Discharge Database (HDD) and Medicaid claims. Using these data, we calculated statewide AHT annual incidence rates.

**Results:**

The databases with the highest case capture rates were the ATR and Medicaid systems, both at 51%, followed by HDD at 38%. Combined, the ATR, HDD and Medicaid systems captured 91% of all AHT cases. The linkage and use of the PAHT definitions yielded an estimated sensitivity of 91% and specificity of 98%. During the study period, we detected an annual average incidence of 34.4 cases per 100,000 children aged <2 years (95% CI 25.1, 46.1) and a case fatality proportion of 22% (10/45). Among the AHT cases, 82% were infants. Significant differences (p < 0.05) in AHT were noted by age and race, but not by sex.

**Conclusions:**

In Alaska, applying the CDC PAHT definition to the multi-source database enabled us to capture 49% more AHT cases than any of the individual database used in this analysis alone.

Serious physical abuse resulting in a traumatic brain injury (TBI) has been implicated as an underreported cause of infant mortality (1). Nearly 80% of all abusive head trauma (AHT) occurs among children <2 years of age (2), with infants (age <1 year) experiencing an incidence nearly 8 times that of 2-year olds (3). Using a large national inpatient data set, Leventhal and others found that among children <3 years of age hospitalized with isolated TBI, 23% were caused by abuse (4).

A few notable studies have employed strategies to identify a population-based incidence of inflicted head trauma. Keenan and others conducted a population-based study of inflicted TBI in North Carolina among children <2 years of age and found an annual incidence of 17 per 100,000 person-years. Young age, male sex, young maternal age, non-European American decent and multiple gestation birth were identified as risk factors (3). Another study using a large national database examined skull fractures and TBIs occurring among children aged <36 months and found an abusive TBI annual incidence of 21.9 cases per 100,000 children; among infants, the incidence was 50 cases per 100,000 (4). This same database was used in a previous study using TBI as the only outcome of interest; in this study, the annual incidence among infants varied between 27.5 and 32.2 per 100,000 for the years 1997, 2000 and 2003 (5). In Scotland, researchers have reported an annual incidence of inflicted TBI of 24.6 per 100,000 infants (6).

Using subdural haemorrhage (SDH), New Zealand researchers reported an inflicted SDH rate among infants to be 14.7–19.6 per 100,000 with substantially higher rates among the indigenous Maori population (32.5–38.5 per 100,000) (7). More recently, Fujiwara et al. used ICD-10 code combinations from the Canadian Institute of Health Information Discharge Abstract Database and found a mean incidence for “narrow, presumptive” AHT in infants to be 13.0 and in 13–24 month olds 2.8 per 100,000 person-years (8).

Most previous studies presenting population-based incidence estimations of inflicted TBI or AHT primarily used hospital billing data and relied heavily on the International Classification of Diseases, Ninth Revision, Clinical Modification (ICD-9-CM) codes or required individual medical chart reviews to extract cases. In Alaska and nationwide, the non-standard use of external cause of injury coding or E-Codes to identify abuse and other mechanisms of injury is problematic (9). Previous studies have suggested substantial under-ascertainment by ICD coding alone of child abuse-related fatalities (10–12) and injuries (13–15).

A few studies have implemented or recommended methods to correct this underrepresentation, primarily through enhanced data linkages (13,16–18). For example, Kaltner and associates used multiple data sources including a hospital trauma registry, child death register and medical chart reviews and found an annual AHT incidence of 51.8/100,000 children aged 0–24 months in Queensland, Australia (19).

The Centers for Disease Control and Prevention developed a recommended framework to improve surveillance and monitoring of the occurrence of AHT. This framework includes the use of both an ICD-9 or ICD-10 Clinical Diagnosis Code, an Injury or Abuse Code (see Table I), as well as a standardized definition: “Pediatric abusive head trauma is defined as an injury to the skull or intracranial contents of an infant or young child (<5 years of age) due to inflicted blunt impact and/or violent shaking.” Excluded from this definition are unintentional injuries resulting from neglectful supervision and penetrating trauma such as from a gunshot or stab wounds (20).

**Table I:** CDC ICD-9-CM and ICD-10-CM coding. AHT cases must have both a clinical diagnosis AND injury/abuse code

AHT categorization	Clinical diagnosis code	Injury or abuse code
ICD-9 coding		
Definite or presumptive abusive head trauma	781.0–781.4, 781.8, 800, 801, 803, 804.1–804.4, 804.6–804.9, 850, 851, 852.0–852.5, 853.0, 853.1, 854.0, 854.1, 925.1, 950.0–950.3, 959.01, 995.55**	E960.0, E967, E968.1, E968.2, E968.8, E968.9, 995.50* 995.54, 995.59*
Probable abusive head trauma	All of those above (except 995.55)	E987, E988.8, E988.9
ICD-10 coding		
Definite or presumptive abusive head trauma	S02, S02.0–SO2.1, S02.7–S02.9, S04.0, S06.0–S06.9, S07.1, S07.8–S07.9, S09.7–S09.9, T90.2,T90.5,T90.8–T90.9	Y00, Y01, Y04, Y07.7–Y07.3, Y07.8–Y07.9, Y08, Y09, Y87.1, T74.1, T74.8–T74.9
Probable abusive head trauma	All of those above	Y29, Y30, Y33, Y34, Y87.2

* Exclude cases in the presence of a fall or accident code.

** Does not require injury or abuse code.

The Alaska Surveillance of Child Abuse and Neglect (SCAN) program was initiated in 2008 and is housed in the Alaska Division of Public Health. This system, which links multiple databases initially focused on fatalities resulting from maltreatment, has since expanded to include injuries. Furthermore, no current study has validated the CDC definitions for use at the local level.

This study describes the validation of the CDC AHT definitions and the process of linking multiple sources of information and applying the CDC AHT definition to detect and determine a robust incidence of AHT among children <2 years of age in Alaska.

## Methods

**Fig. 1:**
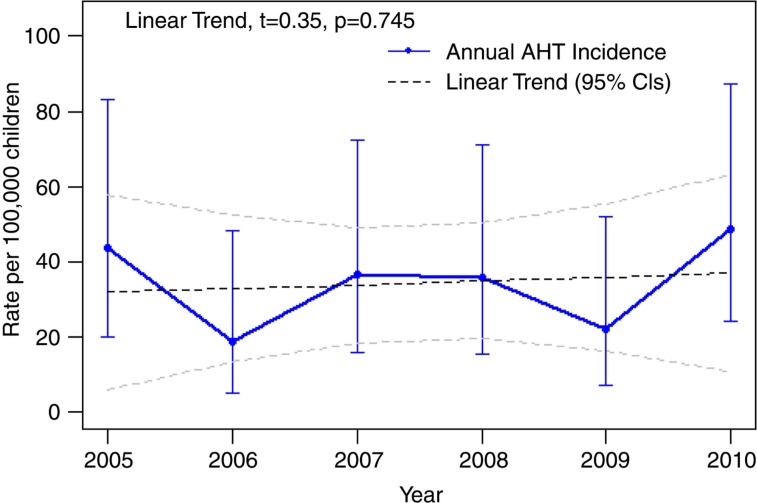
Abusive head trauma rates 2005–2010 in children <2 years of age, Alaska.

Using the Alaska SCAN (21) data system, we extracted AHT cases for children <2 years from 2005 to 2010 using the CDC Pediatric Abusive Head Trauma research guidelines (PAHT, Figure 1) (20). For this assessment, the SCAN system used data from the Bureau of Vital Statistics death records, Alaska Maternal Infant Mortality Review – Child Death Review (MIMR-CDR), Medicaid, Hospital Discharge Database (HDD), Alaska Trauma Registry (ATR) and Alaska Violent Death Reporting System databases. Cases of AHT were identified by applying the PAHT definition to each contributing source independently, and subsequently linked together to identify unique cases.

### Data sources

The Alaska MIMR-CDR committee, established in 1989, reviews all deaths among children <15 years of age. The committee is composed of medical practitioners, topic experts and other professionals. Information from multiple sources including maternal and child medical records, first responder reports, autopsy reports, police reports, court records, vital statistics, child protection and even social media is reviewed to establish consensus on the factors contributing to the death and its preventability. The committee establishes what factor(s) did, probably, possibly, or did not contribute to the death.

The Alaska Medicaid programme includes Denali KidCare, the state's health insurance program for low-income pregnant women, children and teens through age 18. The Medicaid database lists ICD-9 codes for the care billed to recipients.

The Hospital Discharge Data System (HDDS), implemented in 2001 and expanded in 2007, collects approximately 75% of acute care facilities discharges statewide. A number of health facilities are not represented that serve significant portions of the population, which may impact HDDS data completeness (22).

The ATR was started in 1991 and collects information from all 24 of Alaska's acute care facilities. The ATR, based on voluntary reporting from hospital emergency departments, collects information on the most seriously injured patient, on conditions defined by ICD9 diagnosis codes and e-codes (23).

The Alaska Violent Death Reporting System (AKVDRS), established in 2002, is a state-based surveillance system modelled after the National Violent Death Reporting System. Risk factor data concerning violent deaths is collected from disparate sources such as local, state and federal law enforcement agencies and the Office of the Medical Examiner. Case inclusion is based on manner of death and specifically targets homicides, suicides, accidental firearm-related shootings, police-related shootings, terrorism-related deaths and undetermined cause of death (24).

The Alaska Bureau of Vital Statistics is responsible for managing vital records including death data. The death certificate identifies manner of death.

### Linkage

Data were linked in stages between data sets to exclude possible duplicate entry across data systems. The initial linkages between the MIMR-–CDR, Medicaid data, VDRS and vital statistics were performed by matching first name, last name and date of birth. Date of birth and first name were linked using an edit distance format to account for simple typos and transpositions; last name linkage used a Q-grams format to address transposed hyphenated names. All linkage processes between sets required two passes. The first pass used an initial weighting between variables that was highly specific and only captured nearly exact matches. The second pass was more sensitive and required additional hand linkages to capture changes in last names, and other significant variations of identifiers. Furthermore, due to the small numbers of actual cases, all linkages were verified by hand.

Both the ATR and HDD were probabilistically and hand matched based on date of birth, date of hospital admission (±3 days), residence and location of injury. Probabilistic determination was automatically accepted as a match at 95%, hand linked between 75 and 94.9%, and automatically rejected at <75%. All linkages were conducted in FRIL (25).

A total of 6 duplicate cases were identified within three data sets (ATR, HDD and Medicaid), using the PAHT codes. Evaluation of these cases revealed that all within-system (double counts within a single system) duplicates had the 995.55 (Shaken Baby Syndrome) code in the absence of any other code, and there appeared to be subsequent visits for other issues or procedures from the initial incident. Further examination revealed that modifying the PAHT code to require a diagnosis code as noted in Table I or expanded to also allow for eye code diagnosis {361.00 – .05, .10, .30, .33, .8, .81, .89, .9, 362.40, .81} in conjunction with the 995.55 code removed all but 1 of the duplicate cases. However, in this study no effort to recode was needed because of the linkages across systems and use of identifiers, which allowed for all duplicate cases to be detected and removed.

### Validation

Cases of potential AHT among children <2 years of age during the study period were identified, extracted, coded and reviewed by two medical professionals trained in child abuse recognition and evaluation at Providence Alaska Medical Center (PAMC). As the largest paediatric tertiary care centre in the state, a majority of the severe cases of AHT were likely seen at PAMC. Records of children <2 years of age who were seen at Providence during the study period with any of the following ICD-9 coded diagnoses were extracted: 362 (retinal haemorrhages), 431 and 432.9 (SDH), 800–804 (skull fractures), 850–854 (cerebral lacerations/bleeding/contusions), E960–E969 (homicide and injury purposefully inflicted) and E980–E989 (injury undetermined if accidental or inflicted). Cases were coded as definite abuse if any of the following were met: trauma history was inconsistent with the child's injuries or developmental status; pattern of abusive trauma without adequate explanation; intracranial injuries present with other injuries that fit a pattern of abuse without adequate explanation; abuse was witnessed or confessed. All extracted cases regardless of determination were linked (using first and last name and date of birth) with the SCAN system to estimate the reliability and validity of the coding schema.

### Analysis

The final linked database was used to identify both overall and subgroup incidence of AHT among children <2 years of age. Annual incidence was calculated per 100,000 populations. Annual denominators for children <2 years in Alaska were calculated as (annual births+[previous year's births-previous year's infant deaths]).

Subgroup incidence was calculated for infant characteristics, including infant age (<1 year, 1 year), sex (male, female), Race (White, Alaska Native, Asian/Pacific Islander, Other). Subgroup incidence was also calculated for three additional maternal characteristics, maternal age (<20 years, 20–29 years, 30+ years), marital status (married, unmarried) and maternal education (<12 years completed, 12+ years completed). All demographic comparisons were made using median unbiased estimation exact methods to account for small cell sizes with α set at p = 0.05 level. Risk ratios and 95% confidence intervals (95% CI) are also presented. Annual trend was assessed using a quasi-Poisson model to address slight overdispersion in the data.

AHT identification by source is also described using tabular methods. Validity of the PAHT definitions was assessed through a sensitivity and specificity assessment among a subgroup analysis of Providence Alaska Medical Center extracted files.

All analyses were conducted in R, version 2.15.1 (26).

### Institutional review board approval

This evaluation involved linkage of the existing legally authorized administrative databases housed within the Alaska Department of Health and Social Services. Activities conducted by the Surveillance of Child Abuse and Neglect program implemented by the Division of Public Health are outlined in AS Sections 18.15.355–18.15.395, which describes conducting public health activities for issues of public health importance. Under these circumstances of conducting routine public health evaluation, and where no novel data were obtained, Institutional Review Board approval was neither sought nor obtained.

## Results

During the study period, 45 cases of AHT were identified in Alaska among children <2 years of age with an annual incidence of 34.4 cases per 100,000 children (95% CI 25.1, 46.1) and a case fatality proportion of 22% (10/45). Infants accounted for 82% (37/45) of all AHT cases and had an incidence four times that of children aged 12–24 months, and a case fatality proportion of 0.25 (Table II). Among infants, 49% of all AHT had occurred through 3 months of age, 76% through 6 months and 92% through 9 months.

**Table II:** Abusive head trauma characteristics among children <2 years of age in Alaska, 2005–2010

Characteristic	Population*	AHT cases (%)	Incident rate (95% CI)†	Incidence rate ratio (95% CI)	p-Value±
Total	130,683	45	34.43 (25.12, 46.08)		
Infant characteristics					
Age					
1 year	64,561	8 (18)	12.39 (5.35, 24.42)	1	
< 1 year	66,122	37 (82)	55.96 (39.40, 77.13)	4.44 (2.18, 10.36)	<0.001
Missing		0			
Sex					
Male	67,514	20 (44)	29.62 (18.09, 45.75)	1	
Female	63,169	25 (56)	39.56 (25.61, 58.42)	1.33 (0.74, 2.44)	0.338
Missing		0			
Race					
White	79,989	20 (44)	25.00 (15.27, 38.62)	1	
Alaska Native	33,240	11 (24)	33.09 (16.52, 59.21)	1.33 (0.61, 2.75)	0.455
Asian/Pacific Island	10,445	8 (18)	76.59 (33.09, 150.92)	3.10 (1.27, 6.83)	0.015
Other	7,009	6 (13)	85.60 (31.42, 186.32)	3.49 (1.26, 8.25)	0.019
Missing		0			
Maternal characteristics					
Maternal age					
<20	12,982	14 (31)	107.84 (58.96, 180.94)	1	
20–29	75,775	23 (51)	30.35 (19.24, 45.54)	0.28 (0.15, 0.56)	<0.001
30 +	41,896	2 (4)	4.77 (0.58,17.24)	0.05 (0.01,0.17)	<0.001
Missing		6 (13)			
Marital status					
Married	82,205	12 (27)	14.58 (7.54, 25.50)	1	
Unmarried	48,275	29 (64)	59.88 (40.11, 86.00)	4.07 (2.12, 8.33)	<0.001
Missing		4 (9)			
Maternal education					
12+ years	112,748	24 (53)	21.29 (13.64, 31.67)	1	
< 12 years	17,703	13 (29)	73.43 (39.10, 125.57)	3.47 (1.71, 6.73)	0.001
Missing		8 (18)			

* Population=[annual births+(previous year's births–previous year's infant deaths)].

† Incident rates per 100,000 children <2 years.

± p-Value and 95% CI's calculated by median unbiased estimation exact methods.

Relative to White children, Alaska Native children had statistically equivalent rates of AHT (p = 0.74). Asian/Pacific Islander and as well as the “other children” category, however, had statistically increased rates (3.10 and 3.49, respectively). Children born to young mothers<20 years of age had a rate of AHT 3.6 and 20 times as high as 20–29 year olds and 30+ year olds, respectively. Children born to unmarried mothers and mothers with <12 years of education also had significantly higher incidences of AHT compared to the low-risk counterparts (Table II).

During the 6-year study period, the annual incidence fluctuated from year to year, but the overall trend remained flat (Fig. 1).

No single data system identified all AHT or had records for all cases. Among the 3 main sources of information, the ATR captured 51% (23/45) of the AHT cases and had records for 58% (26/45) of the total cases. Similarly, HDD captured 51% (23/45) of the AHT cases and had records for 80% (36/45) of the total cases. Finally, Medicaid captured 38% (17/45) of the AHT cases and had records for 76% (34/45) of the total cases. The two most common causes of missed cases by individual sources were: (a) misclassification or non-specific E-codes and (b) missing E-codes.

**Fig. 2:**
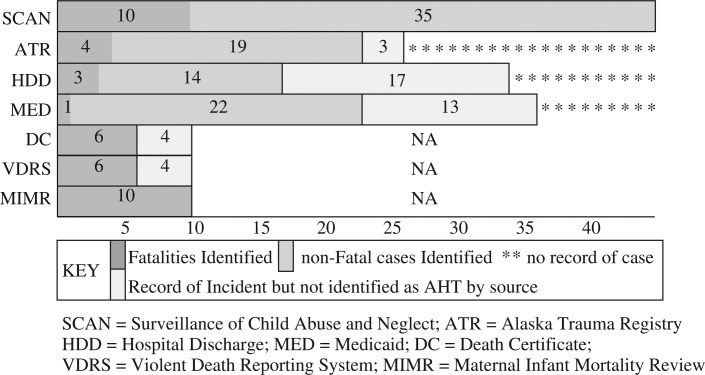
Abusive head trauma detection by source.

The VDRS and death certificate data correctly identified 60% (6/10) of all AHT fatalities. The MIMR-CDR process identified all 10 cases. The most common reasons for being missed by the VDRS and death certificates were lack of intent established and/or perpetrator identified and nonspecific abuse classifications (Fig. 2).

Combined, the ATR, HDD and Medicaid data systems had records for all 45 AHT cases, 38% (17/45) were contained in all 3 sources, 38% (17/45) in 2 sources and 24% (11/45) contained in only 1 of the 3 sources. Furthermore, these 3 systems together correctly identified 91% (41/45) of all AHT cases, with 15% (6/41) being identified by all 3, 22% (10/45) by 2 sources and 56% (25/45) by only 1 of the sources. Among the 4 cases not identified by the ATR, HDD or Medicaid systems, MIMR–CDR identified all 4, VDRS 3 and Vital Death records 3. Both the ATR and Medicaid systems identified the most unique cases (11 each).

### Validation of multi-linkage surveillance

A total of 186 patients met the inclusion criteria for chart review at Providence Alaska Medical Center (PAMC), 34 of which were identified as AHT during the study period by child abuse-trained health professionals. The PAHT algorithm, when applied to the multi-source SCAN data system, yielded a sensitivity of 0.91 (95% CI 0.82, 1.0), a specificity of 0.99 (95% CI 0.98, 1.0), and positive and negative predictive values >95%.

## Discussion

By using SCAN's multiple data sources to identify cases of AHT, we identified 49% more AHT cases in Alaska among children <2 years of age than what would be reported by any single data source alone. Although each data set alone did not have information for all cases, among the records they did have, the most common reason for misclassification in individual databases was missing/miscoded E-codes. Improvement of e-code use and classification by medical providers and medical coders could improve individual source record AHT estimation. The ATR and Medicaid systems each identified the most unique cases of AHT. The ATR only identified 1 unique case record, however among ATR-identified cases it detected a substantially high proportion of AHT cases (88%), whereas all other single systems performed less efficiently. In the State of Alaska, HDD alone only identified 38% of all AHT even though it had records for 76% of the true AHT cases. Most national estimates are based on hospital discharge samples, which are likely to underestimate the true incidence of AHT. In Alaska, even if perfect identification was achieved in the Hospital discharge records, it would still underrepresent the true magnitude by one quarter of the cases. States wishing to implement AHT prevention efforts should seek to accurately quantify the true magnitude of AHT cases through multi-source linkages.

The increased detection of AHT relative to studies based on a single source is most likely due to two main factors: (a) each source has limitations related to types of cases captured (jurisdiction); and (b) the level of precision and detail allowing true identification of AHT. By linking systems together and applying a standardized definition to account for these limitations, we increased the sensitivity of case detection.

The CDC PAHT definition and guidelines performed well in this multi-source linkage setting, with both estimated positive and negative predictive values >95%, an estimated sensitivity of 91% and specificity of 99%. This validation of AHT case detection among the subset of cases seen at Providence Alaska Medical Center supports the extrapolation and use of the PAHT definition to represent a more accurate statewide estimation of AHT. However, further assessment of the PAHT coding during source linkage revealed that some individuals were duplicate and even triplicate counted among some sources. We found this was solely due to the PAHT definition standard allowing the ICD-9-CM code 995.55 (Shaken baby syndrome) to count as a case in the absence of any other code. The 995.55 code appeared to “travel” with the individual for subsequent visits for related procedures, infections and medical maintenance. Caution should be used in interpreting all 995.55 codes as incident cases. We suggest requiring the 995.55 code to be accompanied with a diagnosis code indicated in Table I but also including an eye trauma diagnosis series {361.00 – .05, .10, .30, .33, .8, .81, .89, .9, 362.40, .81}.

Consistent with other research (3,4,18), infants accounted for the majority of AHT cases (82%) with a risk 4.4 (95% CI 2.2, 10.4) times as high compared to 12–24 months of age. Younger infants <4 months account for approximately 50% of all infant AHT, indicating the need for ubiquitous early primary prevention efforts such as Dr. Diaz's Hospital-based intervention and the Period of PURPLE Crying Program. Furthermore, it may be indicated to implement follow-up strategies that attempt to reach high-risk mothers more aggressively through the first 3 months of life (27).

Also consistent with national research, young maternal age was implicated as a strong indicator for child victimization of AHT. Furthermore, unmarried status as well as low maternal education attainment indicate substantially increased risks for child AHT <2 years. These maternal indicators should be incorporated to all AHT prevention efforts in Alaska, including Home Visitation efforts. Finally, minority populations should also receive increased attention, and efforts should be undertaken to determine if current prevention efforts translate across cultural boundaries.

This study highlights the benefits of linking multiple data sources to establish population-based AHT incidence rates. This is consistent with the findings of a previous review that synthesized the literature on the use of ICD codes in child maltreatment research and concluded that using code data alone may under-ascertain CM (9). Over the past decade, improvements have been made in standardizing the use of ICD coding for measuring child abuse (6,14,28). However, it is likely that research limited to ICD coded administrative databases continue to underestimate the incidence of child maltreatment. For example, variations in coding practices among clinicians and personal biases could impact case detection and introduce a misclassification bias (4,5,9) thus limiting overall detection, whereas Keenan et al. reported inflicted TBI incidence only for injuries serious enough to require intensive care (3) which may overlook less serious cases. This study presents findings that may provide researchers, who use only single source data to capture AHT, a context of the extent of potential underestimation.

## Limitations

This study has several limitations that could impact both the generalizability and interpretability of the findings. First, as with any study using ICD coding to identify intent, including intentional injury, misclassification can be introduced. Physicians may be reluctant to provide explicit documentation in the medical record, leading medical coders to miss the physician-assigned diagnosis; additionally, the infrequent use of E-codes can impact the ability to differentiate between abusive and non-abusive injuries. Furthermore, with the transition to electronic medical records physicians may be directly coding the data and have little time to capture the complete diagnosis and contributing factors. Second, these results and the specific methodology employed are potentially only representative of the Alaska population and Alaska data sets. Variation in state data collection systems and hospital coding practices, as well as indigenous populations and geographical makeup, could impact overall generalizability of these findings. Third, some data sets used do not include information from all hospitals or clinics in Alaska. Since some clinics and hospitals serving the military and Alaska Native populations do not contribute to the HDD or ATR, some cases of AHT may have been missed. However, with the linkage of multiple systems the actual amount missed is felt to be substantially less than what an individual data source would have detected. Lastly, infants could be disproportionately represented due to being more vulnerable and potentially having more severe consequences resulting from AHT, which result in medical attention rather than actual differences in AHT occurrence.

## Conclusion

We used multiple data systems to provide a robust statewide estimate of AHT among young children that is higher than previously reported. Clinicians should have a high index of suspicion when evaluating head trauma among young children <6 months of age with young, unmarried or poorly educated mothers, but otherwise should focus primarily on clinical history and presentation. Given that ICD coding is frequently generated automatically by provider diagnoses assigned in electronic medical records, and given the common use of ICD coding for maltreatment research, health care providers should strive to accurately document abuse when it occurs and routinely use e-coding to document external causes for all injuries.
